# Chronic Adhesive Perimetritis

**Published:** 1884-10

**Authors:** James H. Etheridge

**Affiliations:** Professor of Materia Medica and Medical Jurisprudence, Rush Medical College. Gynecologist, Central Free Dispensary, Chicago, Ill.; 1634 Michigan Avenue


ARTICLE II.

CHRONIC ADHESIVE PERIMETRITIS. *By* JAMES H. ETHERIDGE.

*Professor of Materia Medica and Medical Jurisprudence, Rush Medical College. Gynecologist, Central Free Dispensary, Chicago, Ill.*

[*Read before the Chicago Gynecological Society, November*, 1883.]

[CONTINUED FROM SEPTEMBER NUMBER.]

The following disorders are to be excluded if it be possible:

Parametritis,

Hematocele,

Myomas,

Engorgements and deviations of the uterus, and

Fecal impaction.

Chronic oöphoritis.

*From Parametritis.*—In all cases of adhesive perimetritis the free motion of the healthy uterus, upon digital examination, is restricted. This restriction at once leads to exploration of the roof of the vagina. Where the whole roof presents a smooth, hard, resisting mass descending to a uniform depth all around, the probability is always in favor of perimetritis.

Adhesive perimetritis in most cases is readily recognized by the absence of a pelvic tumor, and at the same time by the comparative or absolute immobility of the uterus. The pelvic tumor of parametritis is always readily found, and where it can be outlined above the pelvic brim the presence or absence of cellulitis is made certain. Above the brim, the tumor of parametritis is always adherent to the pelvic wall, whereas that of perimetritis is far enough within the pelvic inlet to allow the fingers to be crowded down between it and the crest of the ilium. Where both affections co-exist, differentiation is impossible.

In parametritis the cervix is always pushed over to one side of the pelvis to make room for the pelvic phlegmon; whereas in adhesive perimetritis the cervix may be centrally located, and the fundus may be drawn or pushed in any conceivable direction, determinable only with the sound.

Immobility of the uterus is a very important point in the diagnosis both of perimetritis and parametritis. In the former, where the adhesions are extensive, the immobility is absolute; while in parametritis it rarely or never happens that fixation is so complete.

In parametritis, where the hyperplasia involves the psoas and iliacus muscles, the retraction of the thigh affords relief from pain; whereas in perimetritis no relief is experienced till both thighs are retracted, and thus the abdominal pressure is removed.

*Diagnosis from Hematocele.*—The anamnesis of hematocele is so different from that of perimetritis that not much trouble exists as to its differentiation. In pelvic blood tumors the onset of the symptoms is terrific; this is sooner or later followed by a season of immunity from suffering, to be followed in some cases by severe constitutional symptoms indicative of pelvic cellulitis and peritonitis, and in others by a gradual but sure return to health.

In chronic hematocele, where the anamnesis is obscure and we are in doubt, resort to the exploring needle is usually decisive.

*From Myomas and Uterine Engorgements and Deviations.*—Conjoined manipulation and the sound usually settle the question of differentiation between fibroids and perimetritis. The same may be said of the diagnosis of pelvic peritonitis and uterine engorgements and deviations.

*Diagnosis from Fecal Impactions.*—Examination per rectum always reveals the presence of impaction therein, and thus enables us to exclude such a cause of obfuscation of the diagnosis.

*From Chronic Oöphoritis.—Practically*, it is useless to differentiate oöphoritis from chronic perimetritis, because where the former exists the latter will almost invariably be found. We can only determine as to the presence of ovarian inflammation by feeling the ovary or by its exquisite tenderness when beyond reach.

*Treatment.*—The treatment of cases of chronic adhesive perimetritis must be determined by the presence of *tenderness*, or by its comparative absence. The treatment of cases characterized by much tenderness would not only be inappropriate for those characterized by little or no tenderness, but would, in the majority of instances, be productive of great harm. Hence it is best to describe the treatment for each class of cases separately.

Before considering this subject in detail, I desire to emphasize, as strongly as possible, the expediency of being governed by the presence or absence of tenderness as to what course we will pursue. Any physician who will do such an unwise thing as to use a speculum and a probe in examining a woman tender from chronic perimetritis will incur the risk of relighting the pelvic inflammation, whose termination no one can foresee. It is a matter, unfortunately, of too common occurrence that patients go home, after a first examination in the physicians' office, to remain in bed several days with severe pelvic pains. I have knowledge of two such occurrences where the result was a protracted illness in bed, terminating in pelvic abscesses. Of course, no amount of plausible explanation can satisfy patients and their friends that such occurrences were not caused by the physician. I think it may be stated, with every showing of good gynecological authority, *that the*

*speculum and probe should not be used in examining patients very tender from previous pelvic inflammations.*

Accordingly, I shall consider those cases that present much tenderness *first*, and *secondly* those cases comparatively devoid of tenderness.

1st. All cases presenting the element of *tenderness*, upon digital examination, should be treated with the sole object of causing its disappearance. Its presence depends upon pressure on the nervous supply of the pelvis by inflammatory exudation, or by congested vessels. Consequently, its treatment is simplified into using such measures as will decongest the pelvic vascular supply. Obversely, whatever will increase the congestion present must be most sedulously avoided.

The following measures are calculated to diminish pelvic congestion:

(*a*) Rest in bed.

(*b*) Hot douches.

(*c*) Analgesics, if pain be present.

(*d*) Sitz baths and pelvic packs.

(*e*) Counter-irritation.

(*f*) Laxatives and tonics.

(*g*) Abdominal bandage.

(*a*) *Rest* in bed should be *absolute.* It should be borne in mind that the valveless pelvic vessels become as easily distended as do the veins of the hand when it depends. The horizontal position, which relieves this vascular distension, should be continued for a period after the patient feels well. At first it should be continuous, the patient not being allowed to leave the recumbent position to take food, to evacuate her bladder and bowels, nor to change her clothes, till she can sit up for hours without resultant discomfiture. Later, she can sit up and move about cautiously for an hour or two; still later, she will be able to be up all day, excepting an hour or two at midday, without experiencing any suffering. When lying down it is well, in very tender patients, to have the knees held up somewhat by a pillow, in order to relax the psoas and iliacus muscles.

It is well to caution against a too protracted lying in bed, as it weakens the heart. Lisfranc had his patients lie in bed a year or longer, for uterine displacements, a practice now fortunately abandoned.

I have often observed the temperature—which was habitually 99° to 100° F. in patients tender from adhesive perimetritis—decline upon putting them to bed for a period of several days. When about to begin the treatment of cases of perimetritis it is well to observe the temperature carefully. The higher it ranges the greater the necessity for enforcing rest. Another valuable practical point to be obtained from thermometry is the length of time to confine the patient to the bed. If I am asked, how long ought we to have the patient remain in bed, I answer, till her temperature remains normal throughout the day, after she has again resumed her usual habits. The length of time necessary to accomplish this varies greatly. Some patients receive all the benefit possible for them to receive in two or three weeks, while others will not obtain a similar result in less than three or four months. A large number of successful treatments will average about one month to each patient, provided the other means of relieving the tenderness be used at the same time.

I have seen many patients recover rapidly who did not use rest, but who used the douches, packs or sitz baths, and blisters. They are the mildest cases known. The severe cases all require rest more or less complete.

*Hot vaginal douches* should be freely used, when borne well. I have now and then seen patients who could not bear them without pain of a very severe degree afterwards. I have often observed that patients who could not tolerate douches, had one or both ovaries prolapsed. *Per contra*, I have much oftener observed that patients with prolapsed ovaries could not only bear douches, but were greatly benefited by them. When douches produce pain and fever, or hemorrhages, they are imperatively interdicted for a time. Their subsequent resumption will, after a time, be warranted, in nearly all cases, by no bad symptoms following them.

Hot water produces vascular tension, which is very soon followed by relaxation. To be satisfactorily effectual, douches should be repeated often enough to *maintain* vascular tension and to permit no relaxation to follow. Hence, they should be repeated every four or six hours—thus the patient can receive from three to five douches daily.

When patients do not remain in bed continuously, it is important to direct them to lie down, well covered, for at least an hour after each douche, to prevent taking cold.

The rapid disappearance of exudations under the use of hot douches is truly wonderful in many cases. I have often seen them disappear in three or four weeks.

The *method* of administering vaginal douches should always include one important point, viz: the impingement of the water against the vaginal vault. They should be administered so as to produce the least amount of fatigue and inconvenience. It is astonishing how awkward many women are in using them.

To use them, with the patient on her back with her hips slightly elevated, requires a reservoir with its outlet tube and vaginal nozzle, a bed pan with a side outlet for attaching a tube to convey the water to a pail on the floor. The alleged distension of the vagina and the immersion of the cervix in a lakelet of hot water, while the patient receives the douche, lying on her back, is, in most instances, imaginary.

A very convenient method of administering the douche, is by placing the patient on two chairs, separated four or five inches, with her vulva between them, over a pail. The hot water, in a pail or pitcher, placed on a stand or stove, can be syphoned out without effort on the patient's part, conveyed to the vaginal vault, thence to the pail. This method can be easily and quickly carried out, and without the assistance of a second person, without wetting or removing the clothing. It is the simplest and easiest method of procedure, and for that reason will be the most likely to be faithfully carried out by the patient. A good substitute for the chairs is the modern water closet, which should be used only when the room is well warmed.

Sitting on the *pot du chambre* to receive vaginal douches, is often too productive of aching legs and compressed abdomen to be commended.

Administering the douches while the patient is in a sitz bath is recommended by many writers, without any very well demonstrated advantages to favor it.

The use of medicated waters for hot douches in chronic perimetritis is of doubtful value. The *heat* of these douches is the remedial agent. Its value, in the great majority of cases, is beyond all question.

*Analgesics* are sometimes necessary. Opium is the type of this class of remedies. Cannabis indica is also very effective. Baker Brown strongly urged the use of conium, as especially possessing power to control pelvic pains. Belladonna, per rectum, is much used, and when thus used its effect is often stronger than when used by the stomach, a point important to remember.

The Gross or Brown-Sequard neuralgia pill is often effective. I have used the piscidia erythrina several times without any satisfactory result. Chloral rarely disappoints us in controlling pains, but its objectionable features are serious. Of all analgesics, opium is the most reliable. The deodorized tincture is the least objectionable form in which to use it. Where an unpleasant cerebral idiosyncracy exists against opium, full doses of the bromide of potassium will almost always prove satisfactory in averting the opium's unpleasant action. The resort to opium should extend over as short a time as possible, for fear of inducing the opium habit. I believe that more patients with adhesive chronic perimetritis become opiophagists than the profession has any conception of.

Administered by the rectum, opium disturbs the digestion less than when it is given by the stomach. We must be mindful of the possibility of mechanically disturbing the parts involved in the inflammation by the use of clyster or a suppository, to the point of producing much pain subsequently,—an accident by no means uncommon.

*Hot sitz baths* and *pelvic packs* are of great service in decongesting the pelvic organs.

In very severe cases the sitz baths are administered with more discomfort to the patient than are the packs. The movements of the patient necessary to taking the baths are often productive of pains which it is desirable to avoid as much as possible. The patient should sit in the bath from 15 to 30 minutes, hot water being added every few minutes during that time. Now and then a patient is nauseated and made dizzy in the bath. When such effects are experienced the packs are to be preferred.

The packs are very simple, very easily used, and are nearly always so productive of relief from pains that the patient falls asleep while enveloped in them. The bed being suitably protected from being wetted, by a rubber or oil cloth covered by a quilt and a sheet, the patient is placed thereon with her pelvis enveloped in a woolen blanket wrung out of hot water. A rubber sheet is folded around the blanket, and the patient is covered with bed clothes and allowed to remain one or two hours. Caution must be used in removing the patient from the pack, lest she take cold. They can be used advantageously daily once or twice till they cease to make the patient more comfortable.

*Counter-irritation* is often productive of wonderful relief from pain and tenderness. It can be applied in the way of permanent blisters, flying blisters, or setons.

Blisters, from three to five inches square, or cantharidal collodion, should be applied, alternately, to the vaginal regions. When cantharidal blisters are used, strangury can be best prevented by the free use of diluent drinks. In anemic patients they should be avoided. The patients who are unduly depressed by them shouid not be re-blistered. A blister can be made pyematous indefinitely by the use of savine ointment. Flying blisters are allowed to heal as soon as possible. The choice between permanent and flying blisters must be determined by the patient's general condition. Permanent blisters are a great drain on the physical resources of women. When well borne, they produce good results very rapidly. When not well borne, they induce a condition of anemia and nervous irritability, that is astonishing in many instances.

The discomfort of a blister is so great as to lead the majority of patients to inveigh against them at first, but several days later, when they have experienced the relief that they bring, they will often call for them.

A lesser degree of counter-irritation than blistering is accomplished by the use of croton oil or of iodine. Vesication, continuously maintained for weeks, by the former, is a very mild method. It is not very painful, and inconveniences the patient but little. Enough of the oil, diluted with an equal amount of glycerine or sweet oil, may be used every second, third or fourth day to accomplish the desired end of pustulating. Too severe pustulation, resulting in erysipelatous inflammation is to be deprecated. Occasionally we find patients who will absorb the oil, and its drastic action greatly enfeebles them.

Its effectiveness is so moderate as to leave more to be desired in many cases. Absorption of the exudate and relief from pain do not follow so conspicuously quickly as they follow the use of blisters, or even of iodine. The best that can be said of its use is, that it is not severe, and it does not lay up the patient.

The tincture of iodine should be used night and morning, painted over the lower part of the abdomen, between the *cresta ilii* from the navel to the pubis. Two or three coats of the iodine should be used at each sitting, till the surface is vesicated. Any further use of this agent is then unnecessary, till the tenderness is lessened somewhat, when it can be applied again in from four to fourteen days, till the part is made sore. Thus the continuous application of iodine can be maintained for several weeks with progressively following good results. The tenderness of the uterus on digital examination, and the various pains produced by the pressure of the exudate, gradually disappear as the latter is absorbed.

The supervention of symptoms of iodism imperatively calls for the temporary discontinuance of the use of iodine. It is perhaps as well to inform brunettes that the iodine paintings *may* have a permanent slight discoloration where thus used. I have actually had patients refuse to use it upon receiving this information! I have often seen great nervous disturbance follow the paintings after several days, which must be regarded as indicating iodism. I have never seen the mammary atrophy produced which is sometimes accredited to this drug.

Occasionally patients are made restless, irritable and nervous by iodine beyond all explanation. Such women are generally among those who are greatly enfeebled by their sufferings, and the further use of iodine on them is wholly unjustifiable.

The length of time of using iodine should be limited only by its continuance to improve the patient's condition. I am in the habit of informing my patients, at the outset, that they must expect to use it *at least* three months as continuously as it can be borne. Its continuance for six months is a matter of common experience.

I desire to emphasize one point, especially, before leaving this part of the subject—and that is, the necessity of covering the whole lower part of the abdomen with this agent. Painting over a small space—*e. g.*, two or three square inches—will not do much good, and will produce nearly as much inconvenience. Unless we observe for ourselves the extent of the paintings we will very often be deceived by being led to believe that enough surface is covered, when, in reality, not one-fourth as much is painted over as we had ordered.

The seton is another method of producing effective counter-irritation. It is applicable to patients confined to the bed. Its great advantages are its certain derivative action, and the small space that it occupies. It can be inserted in either iliac region, an inch above Poupart's ligament.

A dozen small threads of silk can easily be passed, with a flat curved needle, through a fold of skin pinched up tightly. The pain is but a small matter. The skin should first be benumbed by ether spray. The severe inflammation sometimes produced by a seton can be allayed by poultices. The threads should be tied into a loop, to prevent their being pulled out, and then anointed with basilicon or resinous ointment. Every day or two they should be drawn through the opening so as to embed a fresh portion of the threads. In this way they can be drawn through, back and forth, and the suppuration maintained for weeks or months.

The patients should be informed that a scar will always remain after the seton's removal. The possibility of erysipelas and of exhaustion of the patient's vitality following the setons should be borne in mind.

In some apparently desperate cases the seton seems to be the only effective measure. Under its influence the pains gradually diminish, the reflex oppression to the digestive and nutritive functions is slowly lifted, and the gradual improvement is so pronounced that he who once uses setons is inclined to use them frequently.

The length of time of using them should be limited only by the continuance of progressive improvement.

I wish to add, in the way of a summary on this subject, that, of all measures to promote absorption, relieve tenderness, and diminish pains, I regard effective counter-irritation as the most valuable of any single measure. Substantial and most striking improvement follows its use more conspicuously than it follows any one measure.

(*f.*) Nearly all cases of chronic adhesive perimetritis with tenderness need corroborant treatment. Constipation is present in nearly all cases. Copræmia is produced by it, and we soon thereafter have fecal anemia. The impoverished blood leads to imperfect functioning of all the organs, notably of the alimentary tract, which, in turn, leads to mal-assimilation.

Thus the patient drifts into a vicious method of existing, in which the physician usually finds her.

The best corroborant measures for such cases are laxatives and tonics. A course of one or two months of these remedies will put the patients into a condition of nutritional vigor wherein we can hope to see the necessary absorption produced in the pelvic tissues which leads to recovery—a condition which is *absolutely indispensable* to speedy and certain recovery. Unless we can secure this improved condition of the patients' blood, efforts in the direction of cure will be in vain.

Every physician has his own selection of laxatives and tonics. Whatever he has skill in using to secure daily defecations and an increased appetite will be of most avail to him. Compound licorice powder, euonymin, aloes, cascara sagrado, compound colocynth extract with stramonium, the various laxative mineral waters, physostigmin in 1/8 grain doses three or four times daily, iridin, tamarindus, and many other agents can be used in such manner as to result in naught but advantage to the patient. I have used all of them at times. Some patients cannot take laxatives that others can very easily take. A lady whom I know takes a daily dose of 13 drops of the fluid extract of podophyllum with comfort and efficiency, while her sister has the most violent tormina after using a similar dose of the same drug. By trial one soon learns what will act satisfactorily with individual patients. When the daily dose is found, it can be used till the patient is cured. Proper attention to diet, with the constipated and costive habit in mind, should be given at the same time.

The bitter, mineral and stimulating tonics nearly always benefit. Cinchona, gentian, salix, the various mineral acids, and alcohol in one of its many forms, will be found of great utility in different women.

Cases treated in the manner described will surely be benefited. Some of them need a few of the measures described, while others need all of them. In mild cases, improvement results very speedily, while in others it comes reluctantly. The gynecologist who can learn to use remedial measures in such a manner as to continually improve the patient's strength and general nutrition, and at the same time to use whatever seems to be best adapted to each case to promote pelvic absorption without injuring the patient by too officious activity, will learn to temper his prognosis in new cases in a way that will be gratifying alike to himself and to his patient.

Occasionally cases of adhesive perimetritis will be seen wherein the patients are so reduced in strength, and suffer so much, that all that can be done for them is to make them comfortable with analgesics and packs, and to improve their nutrition till they can endure counter-irritation. Such cases are tedious in the extreme. If no inflammatory exacerbations occur, and nutrition can be successfully promoted, such cases will improve. Many times it takes six, twelve, and even twenty-four months to see any decided progress.

Additional to the foregoing, a course of Vichy water will be found beneficial in restoring to health, perimetritic patients who are lithemic or rheumatic. The granular effervescing salts of Vichy are the most convenient fcr using. The chemical reaction of the animal humors plays a most dominant part in nutrition. The functions of nutrition, respiration, and of vital selection, depend greatly on the chemical reaction of the animal fluids. Excessive gastric acidity, with sour or rancid eructations and regurgitations, tympanites, epigastric tenderness, and a bad taste in the mouth mornings, will be corrected by the use of Vichy salts. The life of enforced quietude led by the majority of these patients tends to the development of lithiasis, which, when found, should determine us upon the use of Vichy water.

The abdominal bandage will be found of the greatest possible advantage, by way of affording gentle and firm support and a sense of comfort to the patient. One should be used that can be laced down the sides. It can be made to fit exactly, being made to order by measurement. I know of no supporting bandage so well adapted as the one known as the "Madame La Chapelle's Health Preserver," so extensively advertised in the current medical journals by a business firm in Boston. When adjusted to patients tender from perimetritis it affords instantaneous comfort, and enables them to move around with unwonted steadiness and certainty. Its good effect is only to steady the abdominal organs and prevent too great motion of them, by gentle and universal compression of the abdominal walls.

*2nd. Treatment of Cases Comparatirely Devoid of Tenderness.*—The disappearance of the exudate is what we aim to accomplish. Patients not subjected to medical authority will often recover from perimetritis. Furthermore, it is an established fact that the better the general health, the sooner will the exudate be absorbed. In this way can we explain why, in some instances, without treatment, the immobility of the uterus is relieved in a few months, and why, in other patients, it persists for years.

The natural history of the disappearance of pelvic exudations furnishes a very interesting pathological study. One is most forcibly impressed with the difference found in examining patients in the acute stage, or immediately thereafter, with its complete uterine anchylosis and "deal board" hardness of the vaginal roof, and the comparative freedom of uterine movement twelve or twenty-four months later—and this in cases not under medical treatment in the interval. I examined a patient this month, whom I first examined four years ago, when I found all the pelvic organs immovably fixed by an old gonorrhœal perimetritis, terminating in abscess, of four-and-a-half years previously. This month I find perfect and painless freedom of motion of all of the pelvic organs. In the meantime, in spite of repeated urgings to submit to treatment, she has had no treatment whatever, except what was necessary to preserve her alimentary functions in good order. She is a woman of an exceptionally vigorous and energetic nature, and yet, notwithstanding her uniformly good health, it has taken her eight years to recover without special treatment.

I have seen many cases wherein these changes have taken place in a few months—in from six to twenty-four months—without special treatment. I have never seen another one so long recovering as the one just mentioned.

In view of the fact that many cases recover spontaneously, it would seem that all that a physician can do is to hasten what nature does for these patients.

Attention is directed to the following means of treatment of perimetritis, additional to those already mentioned:

(*g.*) Local treatment.

(*h.*) Massage.

(*i.*) Postural treatment.

(*j.*) Pessaries.

(*g.*) Local applications to the cervix and vagina will be found of the greatest advantage when properly used. The "iodized phenol" (tincture of iodine one part, and carbolic acid, 95% solution, three parts), first recommended by Dr. Batty, of Rome, Ga., will be found adequate to all needs in this direction.

After the cervix and upper vagina are cleansed as much as possible, the entire mucous membrane visible through the speculum should be painted over thoroughly. Subsequently, a pledget of cotton saturated in the phenol is passed into the uterine cavity as far as it can be passed *without producing suffering*, and allowed to remain for 24 hours or less. It can be easily placed there by being loosely wound around an applicator and held there by the forceps as the applicator is withdrawn. A string attached to it permits its removal in 24 hours. The caution against its being thrust through a tender internal os cannot be too positively enjoined.

Painting the upper part of the vagina with the iod. phenol must be productive of good. It thus presents a surface additional to the cervix and enables us to extend the application of counter-irritation.

Iodized phenol is comparatively painless, because of the local anæsthetic action of carbolic acid. It thus possesses a great advantage over nitric acid and over silver nitrate.

The local action of iodine is most pronounced. If the iodide can promote absorption of exudations, surely iodine should be most energetically efficient in these cases. The gratifying part of it is, the pelvic exudations *do* disappear in most instances under iod. phenol.

In many instances the incipient toxic action of carbolic acid is most unpleasantly noticed in its producing vertigo. Such patients quickly taste the phenol—within five minutes of its use. I have never seen any dangerous manifestation of its poisoning.

After the application, a pledget of cotton should be used to prevent any escape of the liquid from the ostium vaginæ. Should it escape, it will blister the part that it traverses and much suffering upon walking and urinating will follow. I

once had a patient in bed four days from this cause, opiates internally and poultices externally to the greatly tumefied labia being necessitated. The cotton can be removed in an hour with safety.

After phenol applications—two or three days—a compact, cream-white slough of mucous membrane covering is removed by the douches. Its appearance indicates the thoroughness and efficacy of the application.

Local treatment seems to accomplish two ends—it promotes absorption, and thus diminishes the strength of the adhesions, and cures the inflammation of the uterus and diminishes its enlargement. Whether the absorption of the peri-uterine exudation leads to a cure of the uterine inflammation and enlargement or not, is perhaps an open question. I am inclined to believe that the disappearance of uterine fixity is necessary to curing inflammation and hypertrophy. One thing every gynecologist will acknowledge—viz.: that every uterus inflamed, with eroded cervical mucous membrane, fixed and enlarged, is freed from its anchylosis when it is restored to its normal condition as much as our art can restore it.

(*h.*) Massage is a method of treating a fixed uterus first regularly described by Norström in 1876. Its use in some patients is of the greatest possible advantage. Its misuse is productive of renewed inflammations, and thus becomes a source of danger. To distinguish between the two, and to avoid the accidents arising from the misuse of massage, requires much caution. If the rule to avoid carrying this treatment beyond the point of producing *endurable* or comfortable (!) pain be inflexibly followed, no harm will follow massage. If it be carried on to the extent of causing much pain, intending to abbreviate the duration of the treatment, we incur the risk of doing incalculable mischief.

Its chief benefit is to be seen in cases where the uterine immobility is not absolute. Where it is absolute, massage does no good whatever. It is when the uterus is partially movable *only* that massage can be of avail.

The idea of uterine massage may be comprehended in the assertion that its application and results are not unlike those of passive motion of a joint immobile from an old arthritis. Its progressiveness of application is warranted differently in different cases. Some patients will bear rapid progression in its use, while others must be treated with great slowness of progress. Daily use of it should be made when it can be borne. Enough irritation should be produced to promote absorption of the exudate, but *not* enough, to produce inflammation.

When used, the patient should be on her back with the knees drawn well up and the abdominal wall relaxed as much as possible. One or two fingers are introduced into the vagina and behind the cervix. The uterus is then to be freely moved in all directions, upwards, forwards, backwards and to either side, the position of the fingers being appropriately changed in order to effect the various movements. Usually it will be found that movement in some *one* direction is the most painful of all movements, and when it is found, it should be especially avoided. Frequently the only movement possible is produced by the vaginal tract, and when so found it should be repeated till it be possible to effect movement by bi-manual manipulation. The length of time required to reach the bi-manual movements varies infinitely. It can be reached in six or ten treatments in some cases, while in other instances it cannot be reached in less than three months under daily applications of massage.

The length of time necessary to effect the movements herein indicated is very brief—one to three minutes being a great abundance of time at first. Later, as we see that uterine movement is becoming of wider range, it is, in many cases, surprising to see how rapidly progress is made, and how progressively the tenderness diminishes, apparently *pari passu* with the increase of uterine mobility. *Then* it is that longer treatments become rapidly effectual in enabling us to reach bi-manual manipulations.

The character of digital pressure when first applied should be described, perhaps. After reiterated cautions contained in this article, against any violence of application to the pelvic organs, it hardly seems necessary to enjoin the utmust caution as to first manipulations by way of the vagina. The pressure should be *gentle* in the extreme, till pain is produced, when it—pressure—should be so modified as to increase the pain to what may be called a bearable extent *only*, then it should cease. Gradually the extent of pressure can be increased till the fundus can be reached by the other hand.

The to-and-fro movement of the fingers through the ostium vaginæ, simulatory of a synusia, as is wrongly practiced by some persons, is wholly unnecessary. I have experienced a difficulty in inducing some patients who have been thus treated to again submit to the treatment, for reasons obvious to virtuous minds.

The first treatment is always one of considerable anxiety to me, because I never know how well or how badly the patient will bear it. In not a few instances I have had an acute inflammation alighted by massage—hence my anxiety over first treatments. It is a good plan to first treat the patients at home and to have them remain in bed for several hours, leaving an opiate and directions as to poulticing, should symptoms of an acute inflammation follow. In a brief time patients can receive treatment at the office of the physician, without incurring risk. After passing the first stage of treatment by massage—viz.:

the securing of some increased uterine mobility without creating inflammation—the next most desirable thing to secure is the ability to effectively practice bi-manual manipulation. When this is secured, the progress of the treatment will be very rapid. Not only will the exudate be made to disappear more rapidly, but uterine engorgement and menstrual disorders will disappear with the most gratifying rapidity. Patients eat and assimilate better, sleep better, the tone of their nervous system is raised greatly, many increase in flesh, acquire greater powers of endurance, and are quickly restored to health.

After bi-manual manipulation can be practiced, the same movements that were made at first are simply increased and exaggerated to the fullest extent. Naught more can be accomplished. It will almost invariably be the case that patients in whom great improvement in the uterine mobility is secured will declare that they are quite well long before the physician is inclined to stop the treatment.

(*i.*) Postural treatment—as first suggested by Dr. Campbell, of Augusta, Ga., I believe—consists in placing the pelvis higher than the chest, and then admitting air into the vagina. The most convenient way of securing this position is to place the patient on her knees and chest, the thighs being in the vertical position, and the chest and neck lying on the same level that the knees occupy. A hard bed will accommodate the patient in assuming this position. The pelvis being highest, it will be found upon retracting the fourchette a trifle that air is admitted into the vagina, and it becomes distended and elongated to its greatest capacity by the ascent (anatomically) of the uterus towards the diaphragm The uterine movement is made by the traction on it of the abdominal organs through their peritoneal connections. Ordinarily, the physician will experience difficulty in even *reaching* the cervix while the patient is thus postured. The uterine movement, upon admitting air into the vagina, is instantaneous, and is generally accompanied by a sort of air inspiratory sound. The patient can easily assume the position, and, with one finger hooked over the fourchette, retreat it toward the cervix till air is admitted. The movement can be accomplished in five to ten seconds, easily. Immediately afterwards the patient should remain in the genu-pectoral position as long as she can comfortably; afterwards she should remain on the bed without assuming, in the meantime, the horizontal position for an instant.

The postural treatment two or three times daily, aids the other remedial measures wonderfully in some cases. Patients often tell me that the pelvic pains are decreased after this movement. In a few instances the sudden upward movement of the uterus is productive of a sickening pain, for a few seconds, when it passes away. This pain gradually diminishes as the treatment is persisted in. In one instance I found it so severe that the patient could not at that time continue the treatment. After blistering the abdomen and cervix, and hot douches for a few weeks, she was able to renew the attempt at postural treatment and to continue it till complete uterine mobility and a return of her general health followed.

One advantage of this treatment ought to be mentioned, perhaps, although it is obvious—and that is, the effect of replacing pelvic organs that are displaced, as the ovaries, tubes, and fundus uteri. A case comes to mind of a young woman, with partial prolapse of the left ovary, with perimetritic adhesions, who suffered intolerably from headaches. After eliminating all other causes of the headaches, it was decided that the displaced ovary was the organ at fault, and a pessary prescribed. She stoutly refused to allow the use of the instrument, and the difficulty was finally compromised by her accepting the postural treatment in lieu of a support. Almost from the first day of the treatment her headaches decreased, and finally disappeared permanently. The elongation of the perimetritic adhesions and their disappearance by absorption, enough at least to relieve the ovarian irritation, is doubtless what followed the postural treatment.

(*j.*) Pessaries and supports have been discussed so much that I speak of them with the utmost reluctance. Many physicians of large gynecological experience use them very often, while other physicians of equally large experience rarely use them.

I wish to dispose of this portion of the subject in as few words as possible. I have but one rule that governs me in using pessaries, and that is, to adjust them as accurately to the displacement as possible and allow them to remain, *provided they make the patients more comfortable than they were without them.* If their effect is negative, or if they produce *any suffering,* I remove them at once. When I say that I " adjust them as accurately to the displacement as possible," the whole ground of pessary adjustment is included. It is on their adjustment that so much discussion and disagreement have arisen about pessaries. Adapting a pessary satisfactorily, presupposes, on the part of the physician, a complete knowledge of the position of the uterus, of the support upon which the pessary is to rest, of the changes that the pelvic organs undergo when the patient changes her position from the horizontal to the vertical positions, and of the allowances to be made for these changes.

When the perimetritic adhesions are so extensive as to produce complete uterine anchylosis, pessaries are of no benefit whatever. It is in cases of partial anchylosis only that they are of advantage. When bearable, they act advantageously by favoring the elongating of the adhesions, and in this way allow more freedom of action of the pelvic organs. It will very often be found that pessaries of gradually increasing length of the longer axis can be successively used as the adhesions become more and more elongated.

Dr. Bozeman introduced a method of elongating pelvic adhesions, which is so simple and so efficient that it ought to be more widely known. It consists of "columning the vagina" with cotton supports while the patient is in the genu-pectoral position. The cotton support rests against the pubic arch and the perineum. It is allowed to remain thirty-six hours, and is then removed by the patient and douches are used. In twelve hours the "columning" is renewed, till the uterus is raised to its normal level. He says that in this way he can raise the uterus two to three and one-half inches, and afterwards the ovary is restored to its normal position, and a Hodge pessary can be easily worn. It should be added that during this "columning" treatment, other means, as iodine and hot water, should be properly used.

I can speak commendably of Dr. Bozeman's method of treatment with the greatest emphasis. Cases of uterine prolapse (partial) with incomplete anchylosis can, in nearly all instances, be improved with this treatment.

Among other agents remedial of chronic perimetritis, pregnancy may be enumerated with much showing in its favor. Where it can be secured, and we can feel assured that its complete consummation can be had, it should emphatically be seriously considered in the married woman.

It is, without doubt, in cases of moderate perimetritis acquired in girlhood (after puberty) where we see so very many women who tell us that they were always "sickly" till after their first confinement, that we find pregnancy has cured the evils resulting from chronic adhesive perimetritis.

CONCLUSIONS.—(*a.*) The changes in the uterine circulation are of the gravest importance. The more I see and treat cases of perimetritis, the more I am convinced that gynecologists will, sooner or later, accept the following deduction as the truth regarding the vast majority of cases of uterine inflammations:

*In the vast majority of cases, perimetritic adhesions, compressing the broad ligaments, retard the venous flow of blood from the uterus, and thus cause uterine congestions, which sooner or later, terminate in inflammations, of varying degrees of intensity.*

Virgins, who enjoy perfect uterine health up to a certain menstruation, where the common cause of suppression, partial or complete—viz., a cold—is in operation, will in very many instances suffer subsequently at every menstruation. In some cases, naught beyond a slight, colorless leucorrhœa will be seen for periods, varying from one or two up to all subsequent menstruations. In many, naught beyond a slight leucorrhœa will be noticed just preceding and succeeding the flow. Still others will experience pains with the menses never before known. In still others, the more serious symptoms will supervene in due course of time, *pari passu* with the progressive increase of the inflammatory developments in the uterus. Duncan expresses the opinion that, next to endocervicitis, perimetritis is the most frequent of all gynecological disorders. I would suggest that the majority of cases of endocervicitis have perimetritis as their causation. Exactly why the mucous membrane of the cervical canal should take on an inflammation in virgins, without an antecedent cause, is a very difficult thing to explain. *Without exception, every single case of endocervicitis that I have seen in the past few years, has had in its*

*anamnesis a menstrual period wherein the flow has been checked partially or wholly, after which the patient was never quite so well.* From that period the symptoms have developed with varying degrees of intensity and rapidity up to the time of medical advice being sought. M. Bernutz, in his remarkable monograph on pelvic peritonitis, nearly thirty years ago, expressed the conviction "That the future knowledge of uterine pathology is as certainly subordinate to an acquaintance with this affection (perimetritis) as pulmonary pathology has been to a complete knowledge of inflammation of the thoracic serous membrane." That later gynecologists seem not to attach more importance to this subject than their writings indicate, is to me a source of profound astonishment.

(*b.*) The large majority of uterine and ovarian displacements will be found complicated with adhesive perimetritis. Every gynecologist recalls the readiness with which the luxated and flexed uterus returns to its displacement after artificial reposition. He also recalls how many cases he finds wherein reposition is totally unjustifiable, because of the violence necessary thereto being liable to light up a new perimetritis or cellulitis.

The presence of extensive perimetritic adhesions, with their resultant uterine anchylosis, comparative amenorrhœa and sterility, will lead the thoughtful gynecologist to abandon the exceedingly common, routine practice of limiting his treatment to cervical applications through the speculum, and to institute every possible means of promoting absorption of the offending exudate. His practice will be wholly inadequate without it. The very many cases of failure to relieve uterine inflammations undoubtedly have, in a great majority of them, an adhesive perimetritis propagating the inflammatory condition persistently and indefinitely, and, thus, because unrelieved, remain the same after as before the institution of treatment.

BIBLIOGRAPHY.—Boivin, Recherches sur * * * * * avortement. Paris, 1828.

Rouchoux, Archiv. Général. de Méd. Paris, 1833.

Grisolle, Hist. des tumeurs phleg. de la foss. iliaq. Paris, 1839.

Bourdon, Tumeurs fluctuantes du bassin. Paris, 1841.

Churchill, Inflamm. and Abscess of Uterine Appendages. Dublin, 1843.

Doherty, On Chronic Inflamm. of Uterus after Labor. Galway, 1843.

Marchal de Calvi, Des Abces phleg. intrapelviens. Paris, 1844.

Valleix, Union Méd., No. 153. Paris, 1853.

Bennet, On Inflammation of the Uterus. London, 1853.

Aran, Leçons Clin. sur les malad. de l'Utérus. Paris, 1858.

Gallard, De l'Inflam. du tiss. cell. qui environne la Matrice. Paris, 1855.

Becquerel, Des. Mal. de l'Utér. et de ses Annexes. Paris, 1859.

Pirigoff, Anat. Typograph. Petropoli, 1859.

Huguier, Allongements Hypertroph. Paris, 1859.

Nonat, Traité des Maladies de l'Utérus. Paris, 1860.

Klob, Patholog. Anat. d. Weibl. Sex, 1862.

Virchow, Archiv. f. Path. Anat. u. Phys. Berlin, 1862.

Bernutz, Pelvi-Peritonite et de ses Diverses. Paris, 1862.

Duncan, Parametritis and Perimetritis. London, 1868.

Brandt, Mal. des Organ. du bassin. Stockhölm, 1868.

Noeggerath, Die latente Gonorrhœ im weibl. Geschl. Bonn, 1872.

Brown, Am. Jour. Med. Sciences. Pelvic Peritonitis. Phila, 1872.

Simpson, Diseases of Women. Perimetritis. Edinburgh, 1872.

Savage, Female Pelvic Organs. London, 1876.

Norström, Trait. des Mal. des Fem. au Moyen de la Méthode du Massage. Paris, 1876.

Barnes, Diseases of Women. London, 1878.

Mundé, Diff. Diag. bet. Pelvic Peritonitis and Pelvic Cellulitis. New York, 1876.

Olshausen, On Puerp. Parametritis and Perimetritis. New Sydenham Soc., 1876.

Spiegelberg, Exudations in Neighborhood of Fem. Pelvic Organs. New Syd. Soc., 1877.

Courty, Maladies de l'Utérus, etc. Montpelier, 1879.

Schroeder, Handbuch der Krankh. der weibl. Geschlechtsorg. Leipzig, 1879.

Bandl, Die Krankh. der Tub., der Lig. u. Beckenperitonäums. Stuttgart 1879.

Griffith, Perimetritis and Parametritis. Saint Bartholomew's Hosp. Rep. London, 1880.

De Sinéty, Gynécologie. Paris, 1880.

Winckel, Pathologie der weibl. Sexual-Organe. Leipzig, 1881.

Tait, Diseases of the Ovaries. Birmingham, 1882.

Fritsch, Handb. der Frauenkrankheiten. Halle, 1882.

Duncan, Diseases of Women. London, 1883.

Heitzmann, Die Entzuendung des Beckenbauchfells beim Weide. Vienna, 1883.

1634 MICHIGAN AVENUE.

